# Sexual Development in *Plasmodium*: Lessons from Functional Analyses

**DOI:** 10.1371/journal.ppat.1002404

**Published:** 2012-01-19

**Authors:** David S. Guttery, Anthony A. Holder, Rita Tewari

**Affiliations:** 1 Centre for Genetics and Genomics, School of Biology, Queens Medical Centre, University of Nottingham, Nottingham, United Kingdom; 2 Division of Parasitology, MRC National Institute for Medical Research, Mill Hill, London, United Kingdom; University of Wisconsin Medical School, United States of America

Malaria is a devastating global disease with several hundred million clinical cases and just under 1 million deaths each year (http://www.who.int/topics/malaria/). It is caused by protozoan parasites of the genus *Plasmodium*, which have a complex life cycle in a vertebrate host and a mosquito vector. Malaria parasites are haploid throughout most of this life cycle, replicating by asexual multiplication twice in a mammalian host: in liver hepatocytes (pre-erythrocytic schizogony) and within red blood cells (blood stage schizogony), and once in the mosquito (sporogony). The essential sexual stage occurs at the transmission from vertebrate to insect. Some asexual blood stage parasites develop into either male or female gametocytes (the precursor sex cells) and following ingestion in a mosquito blood meal differentiate further into gametes in the lumen of the mosquito's gut, where fertilization takes place. The core processes of gametocytogenesis, gamete activation, exflagellation, fertilization, and zygote formation are conserved across the species from the human parasite *Plasmodium falciparum* to the rodent parasite *Plasmodium berghei,* which is an attractive laboratory model, in part because of its ease of genetic manipulation and the shorter time for the maturation and differentiation of the sexual stages. Here, we focus largely on recent functional studies using reverse genetics that have uncovered many aspects of the parasite's sexual development.

## The Onset of the Sexual Phase

In the asexual blood stage of multiplication, merozoites invade erythrocytes, mitotic division produces a multinucleate syncitium (the schizont), and then cytokinesis produces daughter merozoites that burst out and invade fresh erythrocytes. However, a sub-population of intracellular parasites that forego mitosis undergoes gametocytogenesis (the production of gametocytes) in preparation for the sexual phase. The master regulator(s) of this commitment are completely unknown, but gametocytogenesis can be induced chemically or by culture conditions in vitro. The process involves environmental stress responses, depends on parasite density and genetic variation, and is mediated by a number of signaling pathways [1], [2]. Immune factors and the male:female gametocyte allocation as a fitness trait are important in reproduction [3].

**Figure 1:**
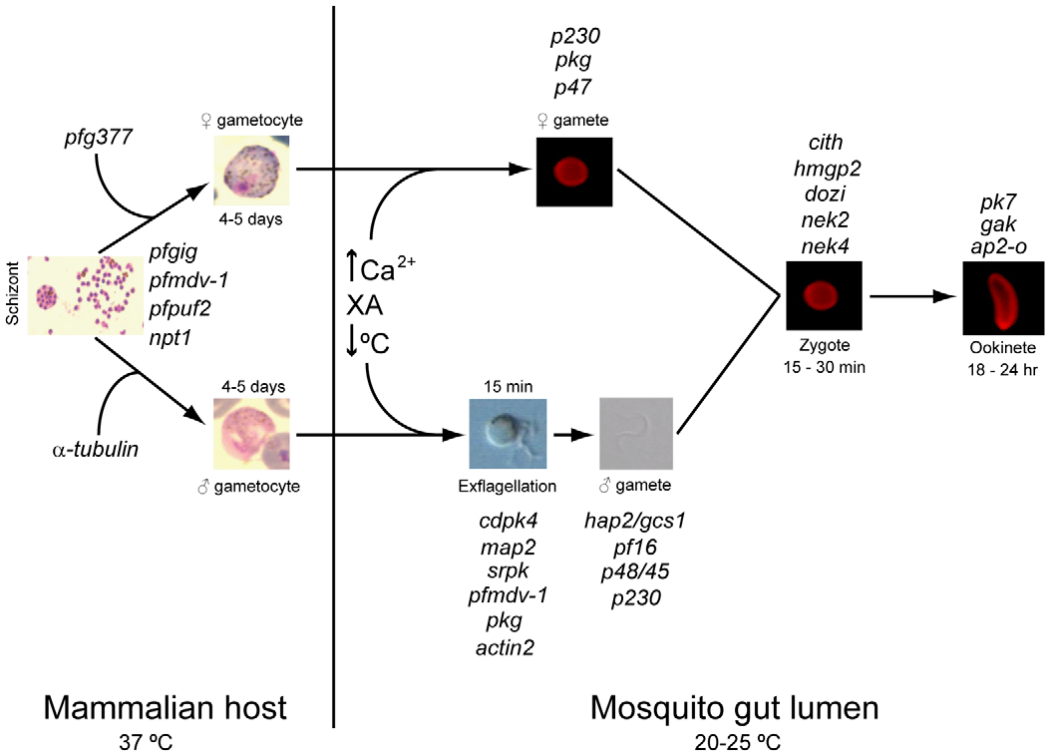
Regulators of gametocytogenesis and the sexual stage of the life cycle. Differentiation of asexual blood stage parasites into gametocytes occurs in the vertebrate host. Ingestion in a mosquito blood meal results in activation of the gametocytes in the lumen of the gut, stimulated by an increase in Ca^2+^, a drop in temperature, and the presence of the mosquito-derived xanthurenic acid, leading to the production of male and female gametes. Fertilization and fusion of these gametes results in zygote formation. Eventual elongation of the zygote leads to the final stage of the sexual cycle, a highly motile ookinete. Genes involved at each stage of the process (as suggested by a number of functional analyses) and time spans for each stage are indicated.

At the molecular level, little is known of the mechanisms by which an individual parasite is triggered to differentiate into a gametocyte. The current consensus is that all merozoites derived from a single schizont are committed to becoming either male or female gametocytes, suggesting that commitment is determined in this previous asexual cycle. Studies using reporters (such as green fluorescent protein [GFP]) controlled by gametocyte-specific promoters (including those for PF14_748, *pfs16, pfg27*, and *SET*) have all implicated the previous schizont stage in commitment to gametocytogenesis [4]. Gender-specific markers introduced using reverse genetics have been observed in sibling progeny from single schizonts that have committed to gametocytogenesis, including *pfg377*, expressed only in females and with a role in egress from the erythrocyte, and *α-tubulin II,* expressed only in males. Gene disruption studies of other putative gametocyte developmental markers in *P. falciparum* (the most important cause of human malaria) include *gene implicated in gametocytogenesis* (*pfgig*) (deletion resulted in reduced gametocyte production) and *male development gene 1* (*pfmdv-1*) (deletion resulted in reduced gametocyte production and defects in male gamete exflagellation) (as reviewed in [5]). More recently, an RNA binding protein, *pfpuf2*, and a novel transporter, *npt1*, were proposed to regulate gametocytogenesis and sexual differentiation [6], [7] (Figure 1). Together, all these studies suggested that there is a gender-specific commitment to gametocytogenesis determined at an early stage in asexual replication. Since sexual development is essential for transmission to the mosquito and completion of the life cycle, this stage is considered to be a major focus for drug and vaccine development to prevent transmission and disrupt the cycle [1], [2].

## The Sexual Stages: Advances in Global “-omic” Studies

Global “-omic” studies of stage-specific gene expression have come a long way. Expression profiling studies (at both transcriptome and proteome levels) have identified many genes expressed in sexual stages, and many of these have a function specific to these stages (see Table 1 of [8] and [5] for details and associated references). These studies identified 200–300 transcripts and corresponding proteins used by *Plasmodium* for and during the production of gametocytes, with a smaller subset of transcripts stored for use after gametogenesis and fertilization. However, although these studies identified stage-specific genes, the unique features that determine the sex of a gametocyte remained obscure.

A proteomic analysis of separate male and female *P. berghei* gametocytes revealed that the two sexual populations are very different at the molecular level [9]. Of the 406 gametocyte-specific proteins identified, only 69 were shared by both sexes. The highest percentage of sex-specific proteins (69%) was found in male gametocytes with large numbers of proteins involved in axoneme assembly and flagellar-based motility, and DNA replication, whereas many of the proteins specific to female gametocytes were for mitochondrial and ribosomal function.

## Lessons from Functional Studies

The advances in genomics, transcriptomics, and proteomics have been complemented by much improved gene targeting technology, providing the potential to undertake systematic functional studies [10]. This approach, together with other functional studies [5], [8], has given us a detailed knowledge of the role of some genes and may provide crucial understanding of the regulation of sexual development in the malaria parasite (Figure 1).

### Male Gametogenesis

Ingestion of the blood meal by the mosquito activates the gametocytes to produce gametes in the midgut. Activation of both male and female gametocytes is due to a drop in temperature, a rise in pH and calcium concentration, and the presence of the mosquito-derived metabolic intermediate xanthurenic acid [11]. For the male gametocyte, activation results in three rounds of rapid DNA replication (within 15 min) and the assembly of flagella, leading to the formation of eight flagellated and highly motile microgametes on the surface of the male gametocyte, a process called exflagellation. Factors important in induction of exflagellation include a rapid rise in intracellular calcium and activation of phospholipase C (PLC) and guanylyl cyclases, leading to increased intracellular cyclic guanosine monophosphate (cGMP), which activates a cGMP-dependent protein kinase (PKG) (reviewed in [12]). Gene disruption studies of the *P. berghei* kinome have revealed a number of essential kinase regulators of male gamete formation [9]–[11]. Calcium-dependent protein kinase 4 (*cdpk4*), mitogen-activated protein kinase 2 (*map2*), and SR protein kinase (*srpk*) have vital roles in DNA replication and axoneme assembly, cytokinesis and axoneme-mediated motility, and exflagellation, respectively [10], [11], [13]. However, in *P. falciparum*, *map2* is essential for asexual replication in the bloodstream, so there may be species-specific differences in the roles of different kinases [14].

Gene disruption studies have implicated other specific proteins in male gametogenesis and gamete motility and fertility, including those coded by the genes *p48/45*, *pf16, hap2/gcs1*, and *actin II*. Deletion of *pf16,* a gene coding for an armadillo repeat motif (ARM) protein of the flagellum central apparatus, results in abnormal movement and reduced fertility, but does not lead to complete sterility [15]. In contrast, deletion of either *p48/45* coding for a 6-Cys repeat domain protein, or *hap2/gcs1* coding for a conserved plant-sterility gene, results in sterility due to the male gamete being unable either to attach (*p48/45*) [16] or fuse (*hap2/gcs1*) [17] to fertile female gametes. Disruption of *actin II* is shown to affect egress of the male gamete from the host cell [18] (Figure 1).

### Female Gametogenesis, and Zygote and Ookinete Development

As with the male, the female gametocyte is activated by exposure to mosquito factors. DNA replication does not occur but the gamete exits the erythrocyte, allowing the male gamete to attach and fuse. Nuclear fusion in the zygote is followed by DNA replication and meiosis [19], with the zygote developing into a motile ookinete that penetrates the gut wall.

Gene deletion studies have defined a number of genes that are vital to zygote and ookinete development [20]. One major mechanism regulating zygote development is translational repression. Deletion of two components of the ribonucleoprotein complex (which withholds certain mRNA species from translation to provide coding potential for proteins during early post-fertilization development) called *dozi* (development of zygote inhibited) and *cith* (homolog of worm CAR-I and fly Trailer Hitch) resulted in hundreds of normally translationally repressed transcripts to be targeted for degradation, with deleterious effects on zygote formation [21], [22]. Two Never in mitosis/*Aspergillus* (NIMA)-related protein kinases (*nek2* and *nek4)* have also been shown to be vital for meiosis in zygote development and ookinete maturation. Other protein kinases that play a role in ookinete development and maturation include those coded by the genes *gak* and *pk7* [10]; however, their mechanism of action is unknown (Figure 1).

## Sexual Stages: Possible Targets for Therapeutic Development?

Functional studies have uncovered a number of candidate targets for drug and vaccine development; however, parasite resistance to current antimalarial drugs will develop, and the first potential malaria vaccine is only in Phase 3 trials. Nonetheless, the sexual stage of parasite development is increasingly being considered to be a key component in future campaigns to block transmission, and to eliminate and eventually to eradicate malaria.

Potential drug targets include proteins coded by genes described in this review, such as the kinase regulators of exflagellation (*cdpk4* and *map2*), regulators of DNA replication prior to meiosis (*nek2* and *nek4),* or PKG. Proteins on the gamete surface such as *p48/45* or *hap2/gcs1* may be vaccine candidates, since antibodies to them induced by immunization could interfere with fertilization. The studies reviewed here are part of the ongoing intensive research to understand sexual development in the malaria parasite with the potential to make a significant contribution in the fight against malaria through the design of intervention strategies for blocking malaria parasite transmission.
